# Supplementary material for: Label-free 3D virtual histology of human formalin-fixed paraffin-embedded (FFPE) prostate needle biopsies with propagation-based phase-contrast micro-CT (PBCT)
Source: bioRxiv. 2026 Jun 7:2026.05.28.728215. Preprint. [Version 2] doi: 10.64898/2026.05.28.728215 (PMC13261793; doi:10.64898/2026.05.28.728215)
Supplement: 1 [file NIHPP2026.05.28.728215V2-supplement-1.pdf]

bioRxiv preprint doi: https://doi.org/10.64898/2026.05.28.728215; this version posted June 7, 2026. The copyright holder for this preprint (which was not certified by peer review) is the author/funder, who has granted bioRxiv a license to display the preprint in perpetuity. It is made available under a CC-BY 4.0 International license.

Supplement

Case ID	Initial Diagnosis	Post-PBCT serial-sectioning (yes/no)
1	benign	yes
2	3+3	yes
3	4+4	yes
4	4+5	yes
5	3+3	no

Table S1: Tissue samples selected for PBCT

<table>
<tr><th>Modality</th><th>Nuclei</th><th>Cytoplasm</th><th>Lumen</th><th>Primary source of signal</th></tr>
<tr><td>Hematoxylin</td><td>blue/purple</td><td>minimal</td><td rowspan="2">white</td><td>Dye-mordant complex binds anionic moieties of DNA/RNA</td></tr>
<tr><td>Eosin</td><td>minimal</td><td>pink</td><td>Anionic dye binds cationic moieties of proteins</td></tr>
<tr><td>PBCT</td><td>white</td><td>light gray</td><td>gray</td><td rowspan="2">Phase changes (δ) induced by variations in electron density; absorption (β) governed by Beer's law

δ/β >> 1 with hard X-rays (>10keV)</td></tr>
<tr><td>Inverted PBCT</td><td>dark</td><td>gray</td><td>light gray</td></tr>
</table>

Table S2: Tissue contrast key in histology and PBCT

bioRxiv preprint doi: https://doi.org/10.64898/2026.05.28.728215; this version posted June 7, 2026. The copyright holder for this preprint (which was not certified by peer review) is the author/funder, who has granted bioRxiv a license to display the preprint in perpetuity. It is made available under a CC-BY 4.0 International license.

**Sample**	**Grade**	**Link**
1	benign	CT<br>https://cephalopod.team/histotomography/Octo9/Neuroglancer/?j=JRW004_invert_z31Histo_match_v1.15_nocontrols<br>Histology<br>https://chenglab.io/osd/daphioMirror/anatomy/histology/?t=JRW004&z=31&c=0.2824365653992985,0.3848448767286696,0.3110399999999999,0.19436481447963797
2	3+3	CT<br>https://cephalopod.team/histotomography/Octo9/Neuroglancer/?j=JRW005_invert_z28Histo_match_v1.15_nocontrols<br>Histology<br>https://chenglab.io/osd/daphioMirror/anatomy/histology/?t=JRW005&z=28&c=0.5917754039605937,1.3961940698475308,0.2705428066888435,0.1674178235027779
3	4+4	CT<br>https://cephalopod.team/histotomography/Octo9/Neuroglancer/?j=JRW006_invert_z10Histo_match_v1.15_nocontrols<br>Histology<br>https://chenglab.io/osd/daphioMirror/anatomy/histology/?t=JRW006&z=10&c=-0.023479754009266385,0.042775547341935584,1.1748974102883647,0.8198543140092132
4	4+5	CT<br>https://cephalopod.team/histotomography/Octo9/Neuroglancer/?j=JRW007_invert_z35Histo_match_v1.15_nocontrols<br>Histology<br>https://chenglab.io/osd/daphioMirror/anatomy/histology/?t=JRW007&z=35&c=-0.10899380611053566,0.11971650827109381,1.2740068917474268,1.8945361105726821

Table S3: Neuroglancer and histology links for sharing

bioRxiv preprint doi: https://doi.org/10.64898/2026.05.28.728215; this version posted June 7, 2026. The copyright holder for this preprint (which was not certified by peer review) is the author/funder, who has granted bioRxiv a license to display the preprint in perpetuity. It is made available under a CC-BY 4.0 International license.

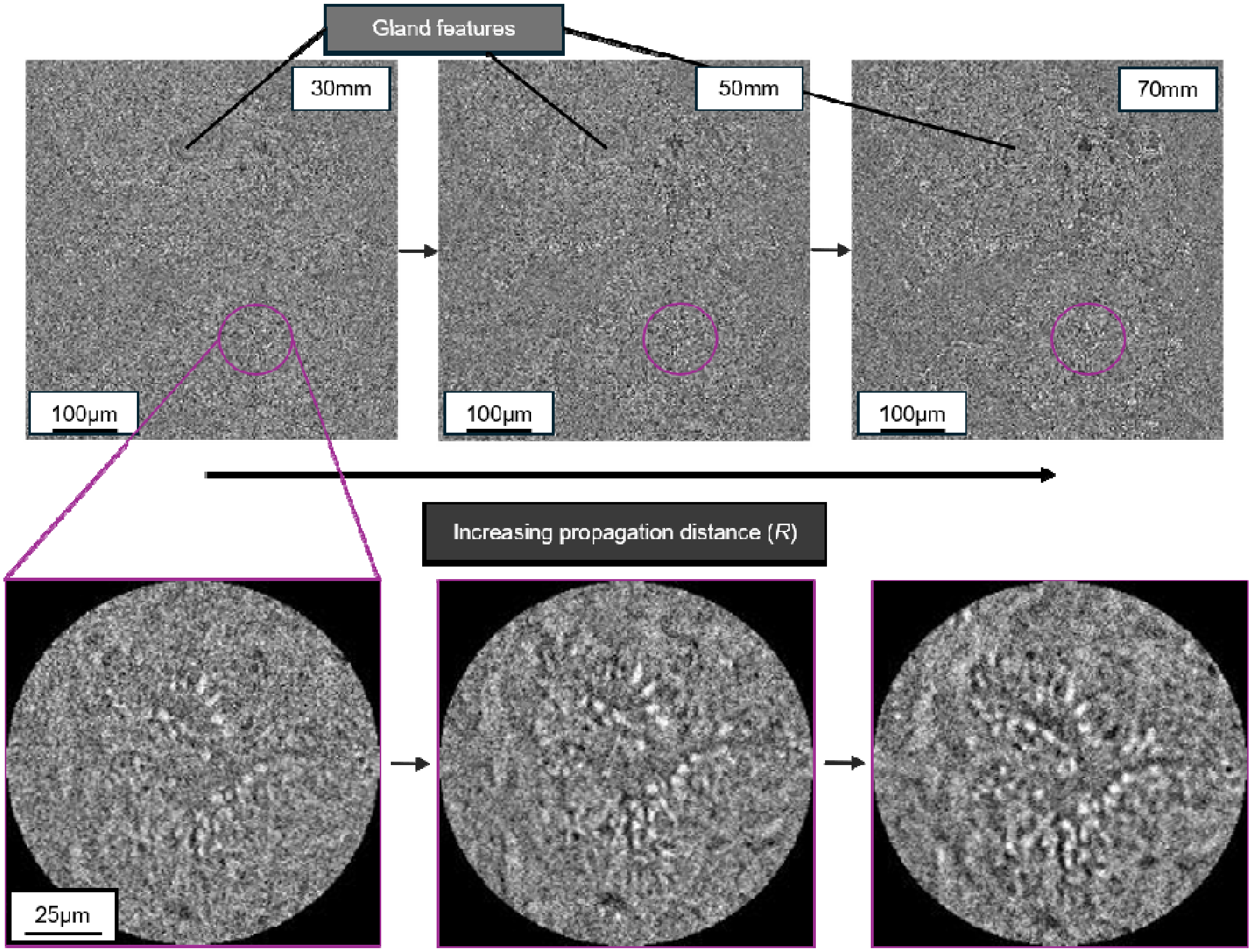

Figure S1: Propagation-based edge enhancement at 30mm, 50mm, and 70mm of propagation distance with 14keV parallel beam X-rays without phase retrieval. (Left to right) 2D slice of the same 3D PBCT scan shown at increasing sample-to-scintillator (propagation) distance. Reconstructions shown here were performed without the use of phase retrieval or a bilateral filter to preserve phase artifacts for comparison. Glandular features and cell nuclei are readily visible at each distance but show significantly improved contrast at 50 and 70mm as compared to 30mm. An example of cell nuclei surrounding glandular lumina are labeled with a purple circle in each snapshot.

bioRxiv preprint doi: https://doi.org/10.64898/2026.05.28.728215; this version posted June 7, 2026. The copyright holder for this preprint (which was not certified by peer review) is the author/funder, who has granted bioRxiv a license to display the preprint in perpetuity. It is made available under a CC-BY 4.0 International license.

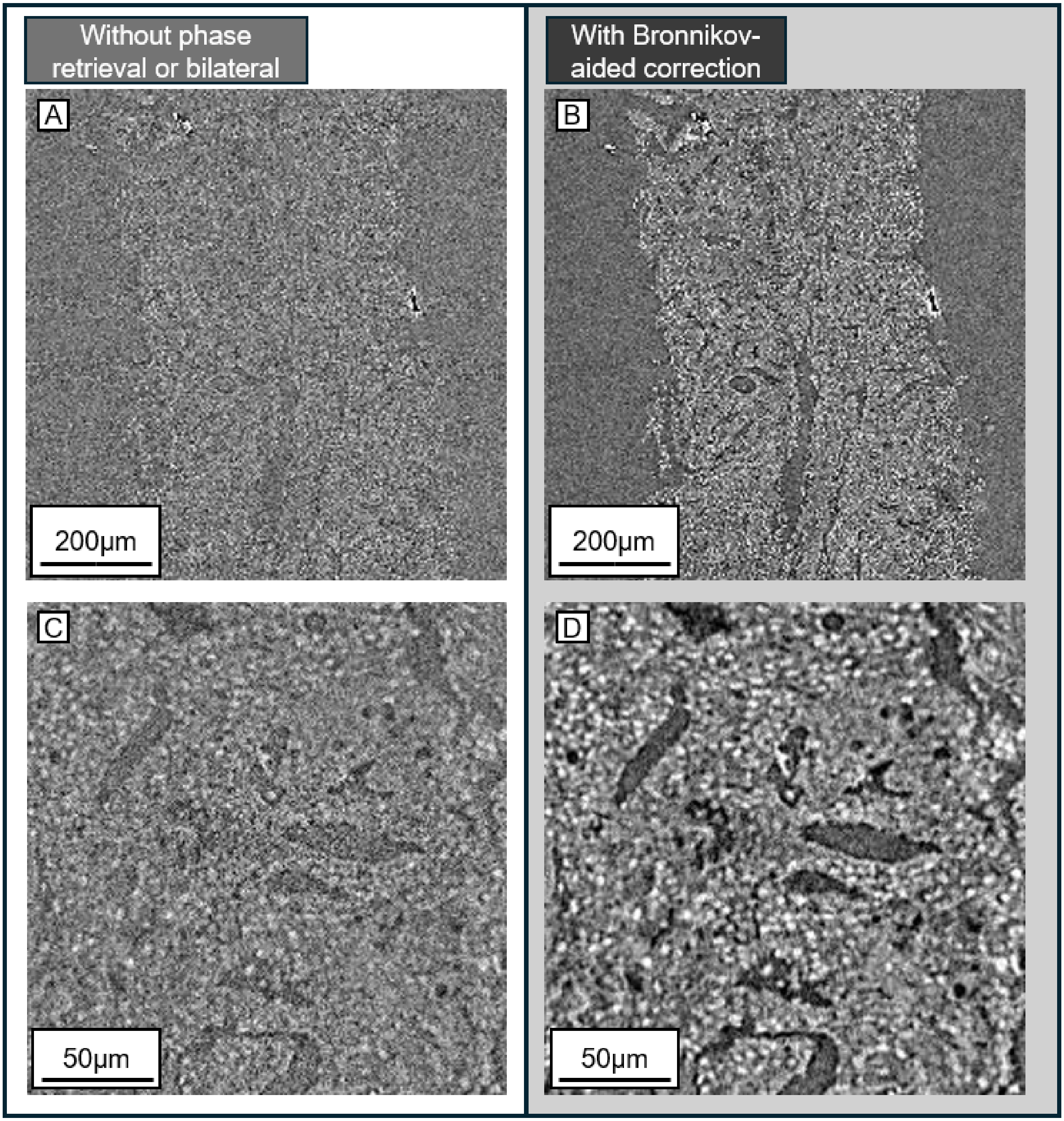

Figure S2: Phase retrieval with Bronnikov-aided correction (BAC) improves contrast while preserving nuclear-scale features. (A) 2D slice of a Gleason pattern 4+4 ROI taken from a scan performed at 50mm propagation distance. Reconstruction of this slice was performed without phase retrieval or bilateral filter. (B) The same ROI from the same sample as (A) reconstructed with the Bronnikov-aided correction (parameter values 2 and 1.25, package = HoToPy) for phase retrieval. (C) Higher zoom snapshot taken from the same reconstruction as (A). (D) Higher zoom snapshot from the same reconstruction as (B)

bioRxiv preprint doi: https://doi.org/10.64898/2026.05.28.728215; this version posted June 7, 2026. The copyright holder for this preprint (which was not certified by peer review) is the author/funder, who has granted bioRxiv a license to display the preprint in perpetuity. It is made available under a CC-BY 4.0 International license.

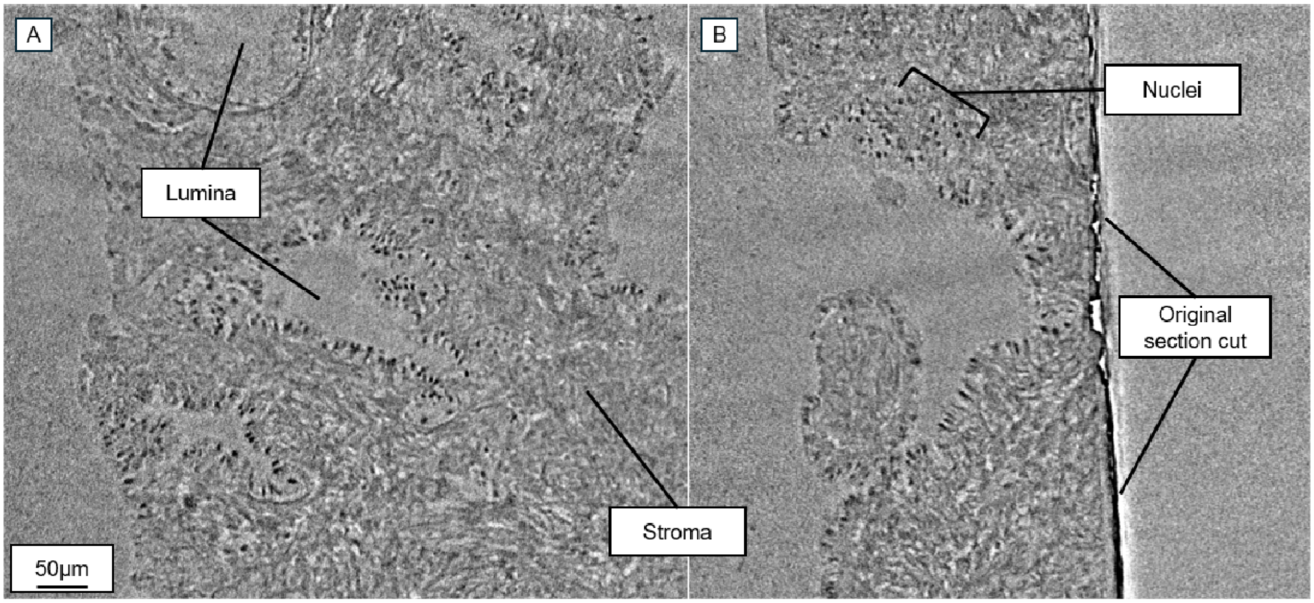

Figure S3: Isotropic resolution of PBCT enables visualization of Gleason grade-defining features in any plane. (A and B) Single (0.65-micrometer depth) orthogonal slices taken from a reconstruction of Case 5. (A) PBCT reconstruction resliced to qualitatively match the plane of initial histologic sectioning. (B) Orthogonal view with respect to (A), highlighting the paraffin edge induced by the microtome and the structures beneath the surface, including nuclear detail.

bioRxiv preprint doi: https://doi.org/10.64898/2026.05.28.728215; this version posted June 7, 2026. The copyright holder for this preprint (which was not certified by peer review) is the author/funder, who has granted bioRxiv a license to display the preprint in perpetuity. It is made available under a CC-BY 4.0 International license.

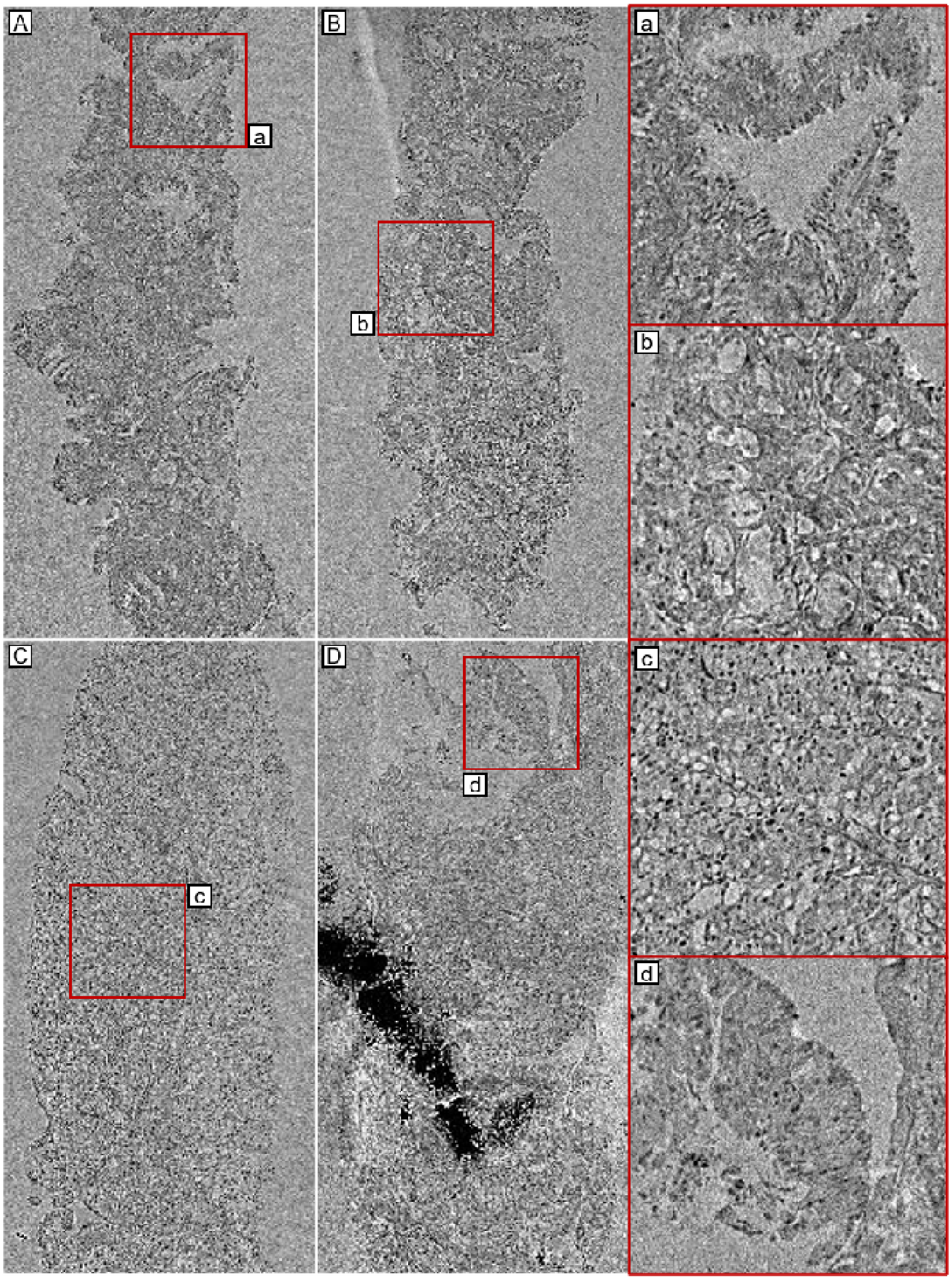

Figure S4: PBCT resolves Gleason grade-defining features from benign tissue to pattern 4+5. (In order A-D) Single virtual-slice ROIs from PBCT needle-core biopsy reconstructions with initial diagnoses of benign (Case 1), pattern 3+3 (Case 2), pattern 4+4 (Case 3), and pattern 4+5 (Case 4). The large patch of high attenuation (dark pixels) captured in D corresponds to calcium phosphate deposits. (a-d) Higher-powered insets selected from regions of each biopsy marked by a red square. (a) Benign gland, (b) cluster of pattern 3 glands, (c) cribriform pattern 4+4 glands, and (d) pattern 4+5 with necrosis.

bioRxiv preprint doi: https://doi.org/10.64898/2026.05.28.728215; this version posted June 7, 2026. The copyright holder for this preprint (which was not certified by peer review) is the author/funder, who has granted bioRxiv a license to display the preprint in perpetuity. It is made available under a CC-BY 4.0 International license.

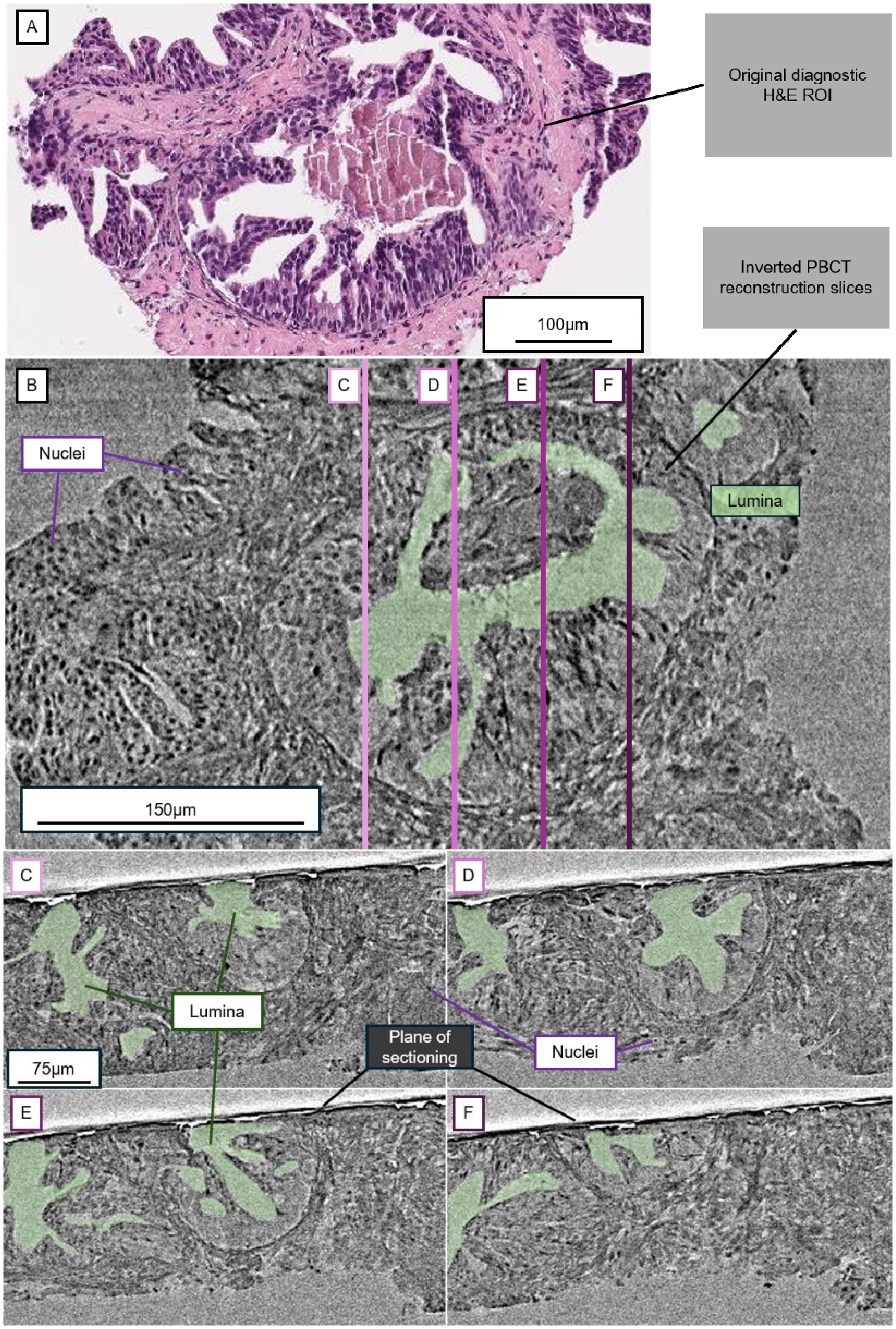

Figure S5: Orthogonal virtual sections reveal variation in prostate gland architecture beneath the plane of sectioning. (A) Virtual single-slice PBCT section of a benign needle-core, cropped to an ROI with features representing prostatic intraepithelial neoplasia (PIN). Glandular lumina and cell nuclei are denoted by green and purple labels, respectively. 4 orthogonal virtual sections separated by approximately 60 micrometers are shown through the gland, denoted by B, C, D, and E. (B) Orthogonal section to (A) illustrating the variation in luminal architecture below what is visible at the cut plane where initial diagnostic slides where sectioned. (C) Additional plane orthogonal to (A), parallel to (B), and ~60 micrometers laterally spaced from (B) and (D). (D) Orthogonal virtual section to (A) ~60 micrometers from (E) illustrating fused glands below the surface of the plane where the initial diagnostic section was cut for this core.

bioRxiv preprint doi: https://doi.org/10.64898/2026.05.28.728215; this version posted June 7, 2026. The copyright holder for this preprint (which was not certified by peer review) is the author/funder, who has granted bioRxiv a license to display the preprint in perpetuity. It is made available under a CC-BY 4.0 International license.

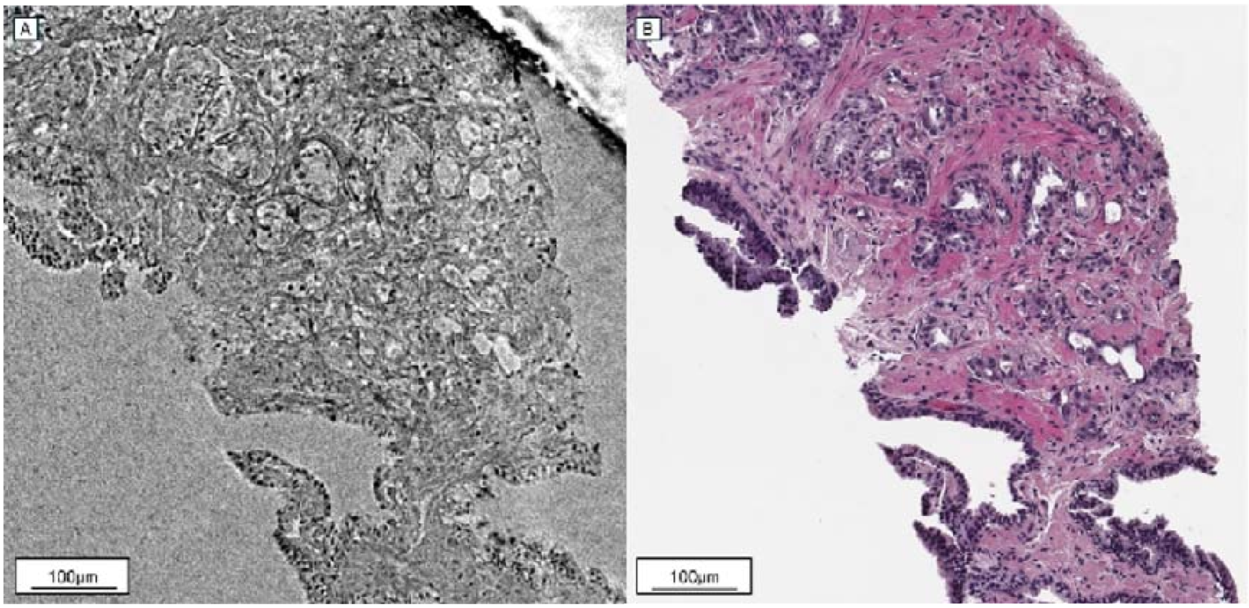

Figure S6: Infiltrative carcinoma visualized by 3D PBCT and serial-section H&E. (A) Single 0.61 micrometer-thick slice taken from a 3D reconstruction of prostate tissue classified as Gleason pattern 3 (Case 2). (B) Correlative H and E-stained histology of the same tissue region captured after PBCT scanning. Crowded round glands consistent with acinar type prostatic adenocarcinoma are visible. Luminal spaces serve as tissue landmarks, and crowded, poorly-formed acini consistent with infiltrative adenocarcinoma are visible across each modality.

bioRxiv preprint doi: https://doi.org/10.64898/2026.05.28.728215; this version posted June 7, 2026. The copyright holder for this preprint (which was not certified by peer review) is the author/funder, who has granted bioRxiv a license to display the preprint in perpetuity. It is made available under a CC-BY 4.0 International license.

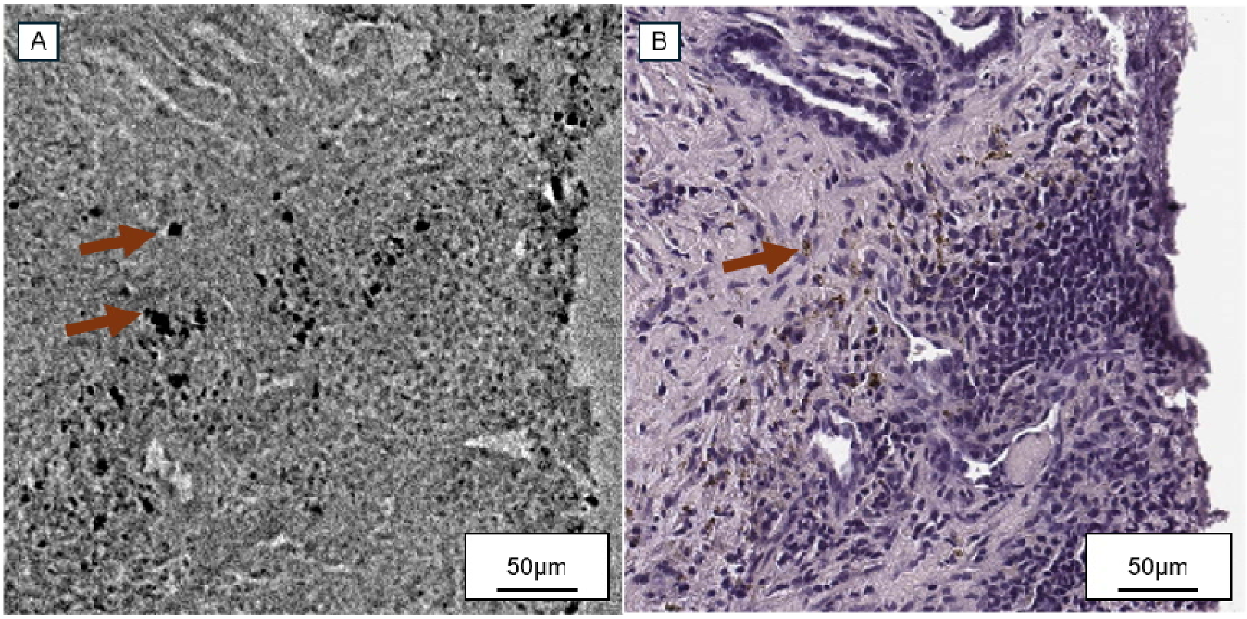

Figure S7: PBCT resolves attenuation consistent with hemosiderin-laden macrophages. (A) Single 0.61 µm-thick slice taken from the reconstruction of case 4, oriented to qualitatively match the plane of post-PBCT correlative histology. (B) Post-PBCT correlative H and E-stained histology of the tissue sample taken from case 4. Hemosiderin laden macrophages are visible as brown/rust-colored deposits (exemplar marked with an arrow).

bioRxiv preprint doi: https://doi.org/10.64898/2026.05.28.728215; this version posted June 7, 2026. The copyright holder for this preprint (which was not certified by peer review) is the author/funder, who has granted bioRxiv a license to display the preprint in perpetuity. It is made available under a CC-BY 4.0 International license.

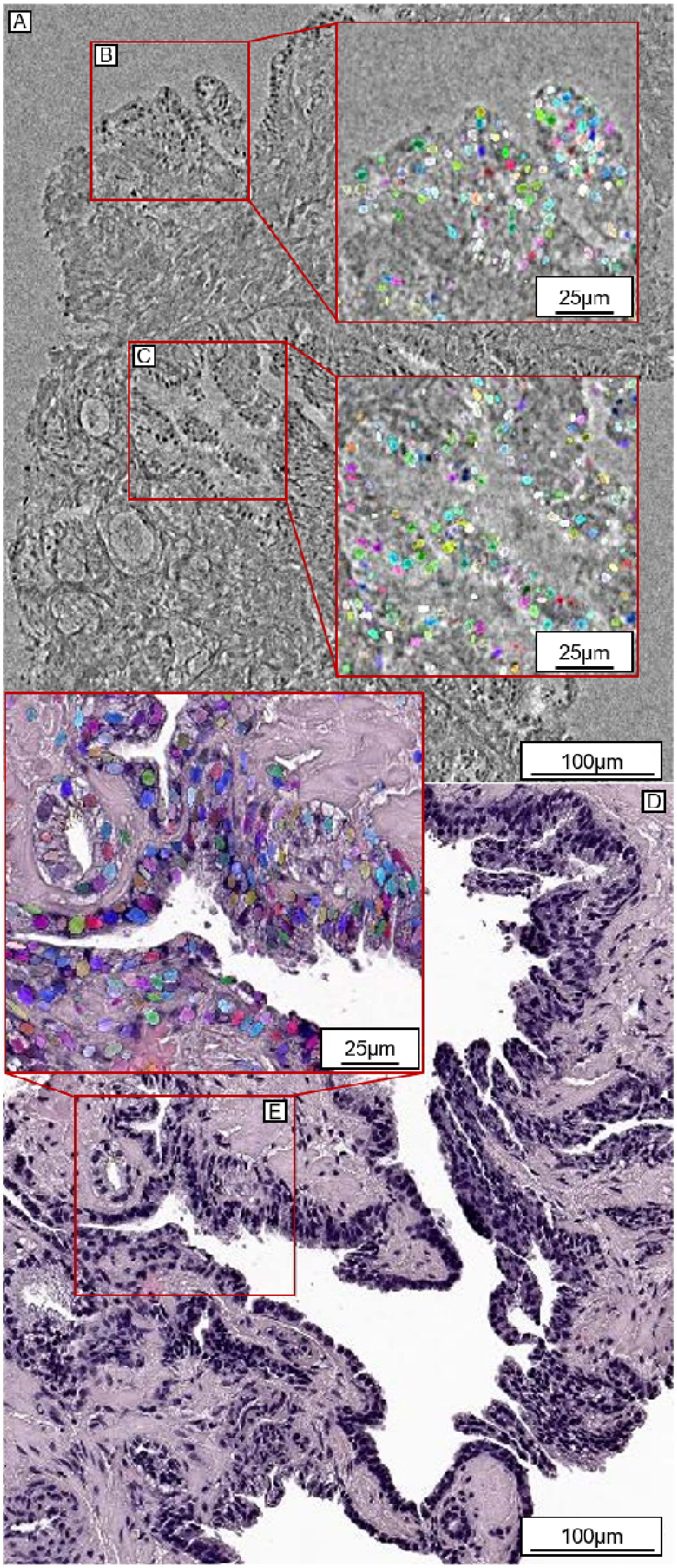

Figure S8: Segmentation of candidate nuclei in 3D PBCT and 2D histology with StarDist. (A) Single-slice ROI taken from a PBCT reconstruction of a pattern 4+4 core biopsy. (B) ROI from the boundary of the core with StarDist 3D U-Net segmentation results rendered as a multicolored instance segmentation. (C) ROI of a prostate gland with StarDist segmentations rendered as in (B). (D) Correlative histology taken from the same core as (A-C) after synchrotron imaging. (E) StarDist 2D U-Net segmentation results rendered as a multicolored instance segmentation as an example of preliminary nuclear detection in correlative histology.

bioRxiv preprint doi: https://doi.org/10.64898/2026.05.28.728215; this version posted June 7, 2026. The copyright holder for this preprint (which was not certified by peer review) is the author/funder, who has granted bioRxiv a license to display the preprint in perpetuity. It is made available under a CC-BY 4.0 International license.

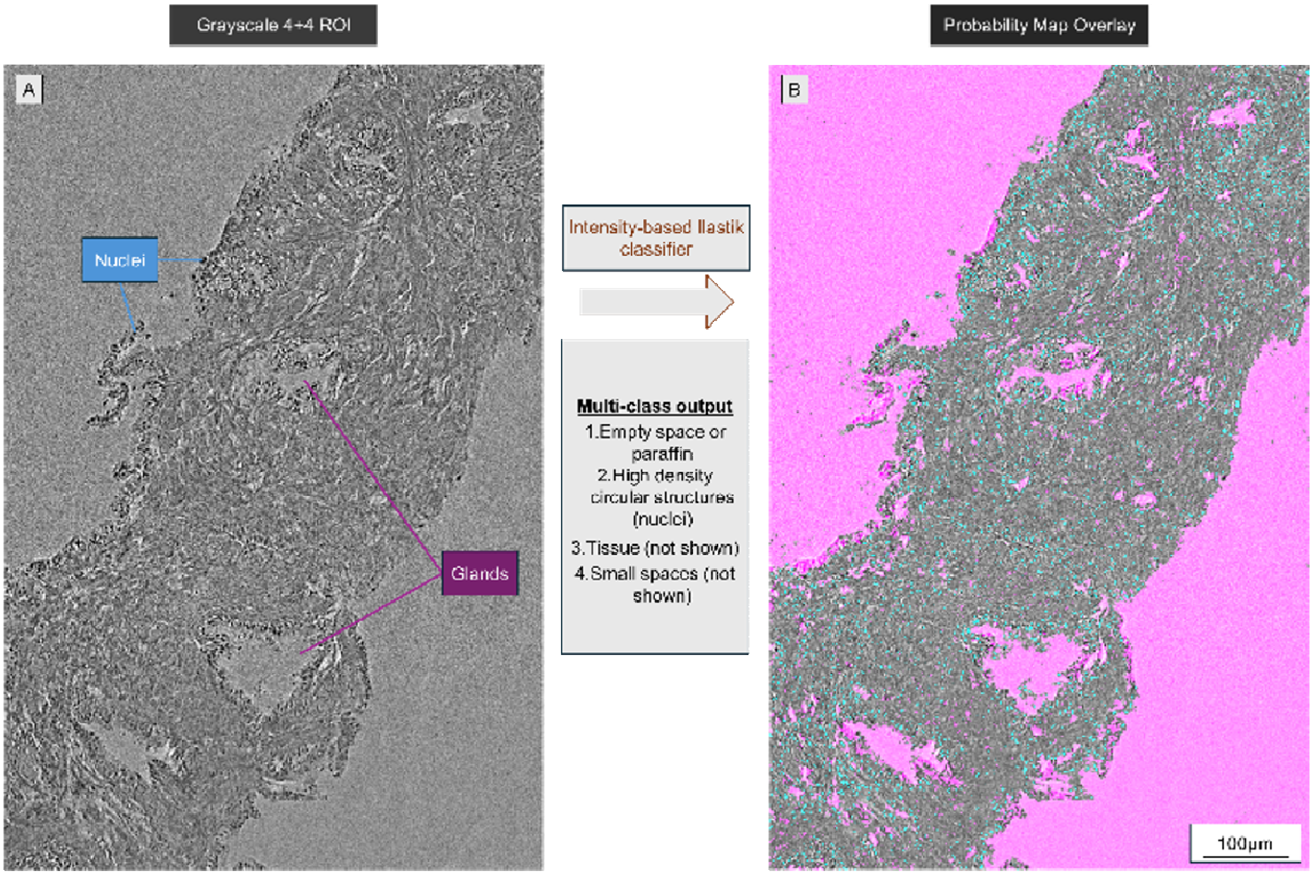

Figure S9. Ilastik random-forest classification segments gland space, tissue, and nuclear structures. (A) Inverted grayscale ROI taken from a Gleason score 4+4 (Case 3, ISUP grade 4) biopsy reconstruction. The 2D slice in (A) was resliced and cropped to mimic the plane of histological sectioning. Nuclei (blue arrow labels, visible as black dots) and glands (magenta arrows) are revealed by PBCT. (B) Ilastik segmentation result visualized as a probability map composite overlay in ImageJ. Nuclei are colored as blue/cyan while glandular lumen and background space are labeled as magenta.

bioRxiv preprint doi: https://doi.org/10.64898/2026.05.28.728215; this version posted June 7, 2026. The copyright holder for this preprint (which was not certified by peer review) is the author/funder, who has granted bioRxiv a license to display the preprint in perpetuity. It is made available under a CC-BY 4.0 International license.

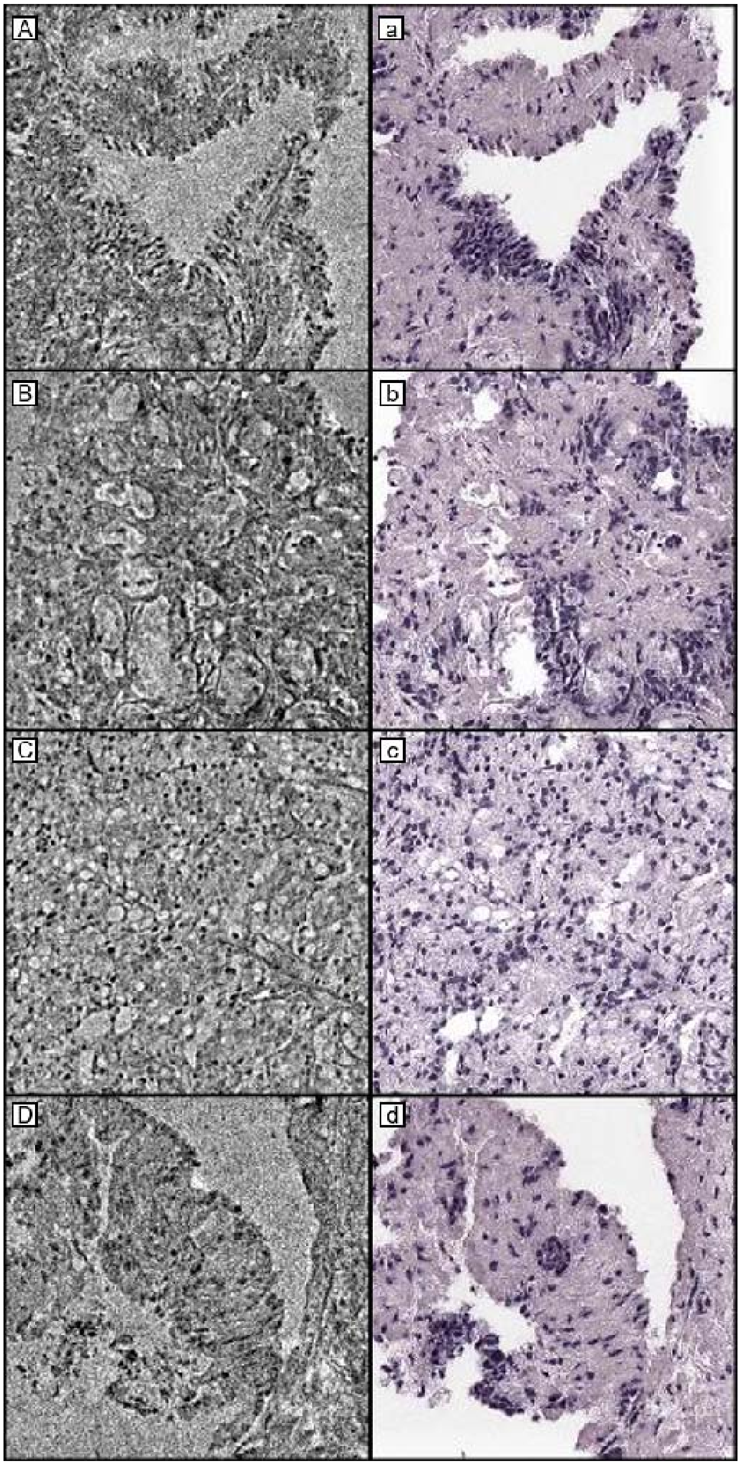

Figure S10: PBCT enables virtual staining (In order A-D) Single virtual-slice ROIs from PBCT reconstructions of Case 1 (benign, A), Case 2 (3+3, B), Case 3 (4+4, C), and Case 4 (4+5, D). (a-d) Corresponding images virtually stained with a cycleGAN trained with snapshots from cases 1 and 3.
